# Impact of Background Noise and Sentence Complexity on Processing Demands during Sentence Comprehension

**DOI:** 10.3389/fpsyg.2016.00345

**Published:** 2016-03-10

**Authors:** Dorothea Wendt, Torsten Dau, Jens Hjortkjær

**Affiliations:** ^1^Hearing Systems, Hearing Systems Group, Department of Electrical Engineering, Technical University of DenmarkKongens Lyngby, Denmark; ^2^Eriksholm Research CentreSnekkersten, Denmark; ^3^Danish Research Centre for Magnetic Resonance, Centre for Functional and Diagnostic Imaging and Research, Copenhagen University Hospital HvidovreHvidovre, Denmark

**Keywords:** effort, processing demands, pupillometry, syntactic complexity, background noise, working memory capacity, reading span, digit span

## Abstract

Speech comprehension in adverse listening conditions can be effortful even when speech is fully intelligible. Acoustical distortions typically make speech comprehension more effortful, but effort also depends on linguistic aspects of the speech signal, such as its syntactic complexity. In the present study, pupil dilations, and subjective effort ratings were recorded in 20 normal-hearing participants while performing a sentence comprehension task. The sentences were either syntactically simple (subject-first sentence structure) or complex (object-first sentence structure) and were presented in two levels of background noise both corresponding to high intelligibility. A digit span and a reading span test were used to assess individual differences in the participants’ working memory capacity (WMC). The results showed that the subjectively rated effort was mostly affected by the noise level and less by syntactic complexity. Conversely, pupil dilations increased with syntactic complexity but only showed a small effect of the noise level. Participants with higher WMC showed increased pupil responses in the higher-level noise condition but rated sentence comprehension as being less effortful compared to participants with lower WMC. Overall, the results demonstrate that pupil dilations and subjectively rated effort represent different aspects of effort. Furthermore, the results indicate that effort can vary in situations with high speech intelligibility.

## Introduction

Speech communication provides a major basis for human interaction. Speech intelligibility has traditionally been measured in terms of the speech reception threshold (SRT) which reflects the signal-to-noise ratio (SNR) at which 50% of the words or sentences have been correctly recognized. However, these measures are typically obtained at low SNRs which do not correspond to everyday-listening situations that typically take place at SNRs of +5 to +15 dB (Smeds et al., 2015). In such more realistic communication situations, despite the fact that speech intelligibility is high, people may experience considerable difficulties when listening to speech. There has recently been growing interest in identifying the factors that cause these difficulties and attempts have been made to characterize the processing demand or processing load (Johnsrude and Rodd, 2015) involved in speech comprehension (Gosselin and Gagné, 2010, 2011; McGarrigle et al., 2014).

Processing demands can be imposed by two factors: *stimulus-related* factors that are associated with properties of the stimulus (e.g., noise degradation or linguistic complexity), and *listener-related* factors that reflect the perceptual and cognitive abilities of the listener [e.g., hearing impairment or working memory capacity (WMC)]. Regarding stimulus-related factors, the degradation of the speech signal due to the presence of background noise has been demonstrated to have an impact on the processing demand (e.g., Rabbitt, 1968; Pichora-Fuller and Singh, 2006). Varying the SNR can thus be used to induce higher or lower processing demand during speech comprehension, such that a higher amount of noise imposes a processing demand. Linguistic aspects, such as syntactically complex sentence structures, have been shown to decrease speech comprehension (Just and Carpenter, 1992), decrease sentence intelligibility (Uslar et al., 2013) and increase the sentence processing duration (Wendt et al., 2014, 2015). Hearing impairment, as a listener-related factor, typically degrades the representation of the speech signal in the auditory system which, in turn, can affect speech recognition (e.g., Plomp and Mimpen, 1979; Wingfield et al., 2006) and the sentence processing duration (Wendt et al., 2015). Moreover, cognitive abilities, such as a person’s WMC, have been related to speech recognition performance (e.g., Lunner, 2003; Akeroyd, 2008). It has been suggested that individual cognitive recourses can be utilized to partly compensate for changes in the processing demand imposed by stimulus-related factors, even though the relationship between cognitive abilities and processing demand remains controversial (Ahern and Beatty, 1979; Verney et al., 2004; van der Meer et al., 2010).

The amount of cognitive resources utilized by a listener in a speech comprehension task can be defined in terms of *effort* (see also Johnsrude and Rodd, 2015). In other words, effort is a measure indicating the amount of resources deployed when processing speech, which depends on the interplay of the processing demand imposed by the stimulus-related factors (e.g., background noise, sentence complexity) and the listener-related cognitive abilities (such as WMC). A person’s effort involved in speech comprehension has been measured using various methods and techniques (see McGarrigle et al., 2014 for a review). Subjective measures, such as perceived effort experienced during speech comprehension, have been tested using rating scales or questionnaires. Rudner et al. (2012) tested the effect of both noise level (in terms of SNR) and noise type (stationary vs. fluctuating) on subjective ratings of the perceived effort experienced in a sentence recognition task. It was found that the subjectively rated effort was affected by both the type of the background noise and the SNR. Although a fluctuating noise masker typically provides a release from masking (e.g., Festen and Plomp, 1990; Wagener et al., 2006), implying increased recognition rates compared to the condition with a stationary noise, listeners rated speech recognition in this noise condition to be more effortful. Rudner et al. (2012) also reported that rated effort increased with decreasing SNR consistent with other studies (Humes et al., 1999; Hällgren et al., 2005; Zekveld et al., 2010). Physiological correlates of processing effort include pupillary responses measured during speech tasks (see Kahneman and Beatty, 1966; Kahneman, 1973; Poock, 1973; Beatty, 1982; Granholm et al., 1996). More recently, there has been an increasing interest in measuring pupil dilations during speech perception in acoustically challenging situations (Kramer et al., 1997; Zekveld et al., 2010, 2011; Koelewijn et al., 2012; Kuchinsky et al., 2013). Zekveld et al. (2010, 2011) reported increased pupil dilations as an index of effort depending on speech intelligibility and type of background noise. Some studies have recorded subjective ratings of effort and pupil dilations in the same listeners (Zekveld et al., 2010, 2011; Koelewijn et al., 2012), but the relationship between the two measures has not yet been clarified. While Koelewijn et al. (2012) showed that the subjective ratings were positively correlated with pupil dilations during a speech recognition task, Zekveld et al. (2010) reported significant correlations between the rated effort and intelligibility but did not find any correlation between the subjectively rated effort and pupil dilations.

Working memory capacity has also been related to both subjective ratings and pupil dilations. Zekveld et al. (2011) reported a positive correlation between digit span test scores (as an index of WMC) and pupil dilations. Moreover, van der Meer et al. (2010) showed that listeners with higher fluid intelligence scores showed larger pupil dilations while performing a difficult task compared to individuals with lower scores. This led to the “resource hypothesis” (van der Meer et al., 2010) stating that individuals with better cognitive abilities, including higher WMC, allocate more resources, leading to a *higher* processing effort as reflected by larger pupil dilations. However, individuals with greater WMC have also been shown to rate listening as being less effortful (e.g., Rudner et al., 2012). This led to the “efficiency hypothesis” stating that individuals with higher cognitive resources report *lower* perceived effort due to more efficient processing (Ahern and Beatty, 1979; van der Meer et al., 2010). In line with this, the ease of language understanding (ELU) model suggests that it is less effortful for individuals with a high WMC to process a distorted speech signal (Rönnberg, 2003; Rönnberg et al., 2013). This seems to be in conflict with the resource hypothesis arguing that individuals with higher WMC engage more cognitive resources leading to *higher* effort. However, whereas the resource hypothesis is based on studies employing pupil response as a physiological correlate of effort, the ELU model refers to studies using subjective ratings as the indicator of effort. Thus, it may be that the two metrics represent different components of processing demand.

The present study attempted to distinguish between the outcomes obtained with rated effort vs. pupil dilation. Here, subjective ratings of effort, termed “perceived effort” (McGarrigle et al., 2014), were considered as an indicator of how effortful the process of speech comprehension is experienced by the participants. In contrast, pupil responses were considered as an indicator of “processing effort”. Perceived effort and processing effort were measured in an audio-visual picture-matching paradigm. In this paradigm, the participant’s task was to match a spoken sentence with a picture presented before the sentence. This paradigm was designed to capture several levels of speech processing involved in the comprehension of speech in background noise. This includes both lower-level perceptual processing, such as the separation of the speech signal from the background noise (Johnsrude and Rodd, 2015) and higher-level cognitive processes including linguistic and syntactic operations, such as a thematic assignment of the characters’ role in the spoken sentence (see e.g., Wingfield et al., 2005). In the applied picture-matching paradigm, a mental assignment of the characters’ roles (i.e., *who is doing something to whom*) is required to accomplish the comprehension task. By employing the paradigm, it was investigated how different levels of the SNR (at high speech intelligibility levels) and the variation of the syntactic complexity of the sentence structure affect perceived effort and processing effort. Furthermore, it was examined how individual participants’ cognitive test scores were related with perceived effort and processing effort.

## Materials and Methods

### Participants

Eleven female and nine male participants with normal hearing participated in the experiment, with an average age of 23 years (ranging from 19 to 36 years). The participants had pure tone hearing thresholds of 15 dB hearing level (HL) or better at the standard audiometric frequencies in the range from 125 to 8000 Hz. All participants performed better than 20/50 on the Snellen chart indicating normal or corrected to normal vision (according to Hetherington, 1954). All experiments were approved by the Science Ethics Committee for the Capital Region of Denmark.

### Stimuli

#### Speech Material

Thirty-nine items from the German Oldenburg Linguistically and Audiologically Controlled Sentence corpus (OLACS, see Uslar et al., 2013) were translated into Danish language and recorded. Each sentence describes two characters and an action being performed by one of the characters. All sentences contained a transitive full verb such as *filme* (“film” in **Table 1**), an auxiliary verb *vil (“will”*), a subject noun phrase *den sure pingvin* (“The angry penguin”) and an object noun phrase *den søde koala* (“the sweet koala”). Each speech item was recorded with two different sentence structures in order to vary the complexity of sentences without changing word elements. Each sentence was either realized with a subject-verb-object structure (SVO I and II in **Table 1**) as well as with a syntactically complex object-verb-subject structure (OVS I and II in **Table 1**). While the SVO structure is canonical in Danish syntax and considered to be easy to process, written and spoken OVS sentences in Danish are more difficult to process (see Boeg Thomsen and Kristensen, 2014; Kristensen et al., 2014).

**Table 1:** Examples of the two sentence structures that were presented in the audio-visual picture-matching task.

Sentence structure	Example							
	Word1	Word2	Word3	Word4	Word5	Word6	Word7	Word8
SVO I	Den	sure	pingvin	vil	_PTD_filme	den	søde	koala
	**The angry penguin will film the sweet koala**							
SVO II	Den	søde	koala	vil	_PTD_filme	den	sure	pingvin
	**The sweet koala will film the angry penguin.**							
OVS I	Den	sure	pingvin	vil	_PTD_den	søde	koala	filme
	**The smart penguin, the sweet hare will film**							
OVS II	Den	søde	koala	vil	_PTD_den	sure	pingvin	filme
	**The sweet koala, the angry penguin will film**							

*All sentences contained eight words and have either a subject-verb-object (SVO) or an object-verb-subject sentence (OVS) structure. The onset of word five is defined as point of target disambiguation (PTD).*

In both (SVO and OVS) sentence structures, the participants need to identify the semantic roles of the involved characters. The role assignment of the character that carries out the action (the agent) and the character that is affected by the action (the patient) is possible only after the auxiliary verb *vil*. Until the auxiliary verb, both sentence structures are ambiguous with respect to the grammatical roles of the involved characters and, thus, no thematic role assignment can be made. The auxiliary verb *vil* is either followed by the transitive verb *filme* (“film” see word 5 in **Table 1**), indicating a subject noun phrase at the beginning of the sentence, or by the article *den* (“the” see word 5 for the OVS I and II), informing the listener about the object role of the first noun. Since word 5 within each sentence provided the information required performing the comprehension task, the onset of word 5 is defined as the point of target disambiguation (PTD) for all sentence structures (see **Table 1**). Care was taken in selecting actions, agents, and objects that were non-stereotypical for any of the characters (for example, baking is a typical action of a baker). This constraint was employed to make sure that the participants did not make premature role assignments based on any anticipation of an agent’s characteristic action.

#### Visual Material

**FIGURE 1:**
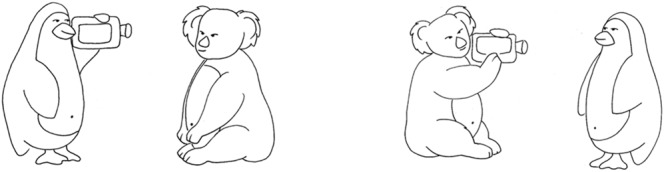
**Example of a visual stimulus pair used in the audio-visual picture-matching paradigm.** The left figure shows a target picture corresponding to the sentences *Den sure pingvin vil filme den søde koala* (“The angry penguin will film the sweet koala”; SVO I in **Table 1**) or *Den søde koala vil den sure pingvin filme* (“*The sweet koala, the angry penguin will film.*”; OVS II in **Table 1**). The right figure shows an example for the corresponding competitor picture of the same sentences. Only one of the pictures, either target or competitor picture, was presented during the paradigm.

Pictures from the OLACS picture set were used, which were created for eye-tracking purposes (see Wendt et al., 2014, 2015). Each sentence was presented with either a target or a competitor picture. The picture illustrating the situation as described in the spoken sentence was defined as target picture (left panel of **Figure 1**). The competitor picture showed the same characters and action but interchanged roles of the agent and patient (right panel of **Figure 1**). Both the competitor and the target picture were of the same size, and within each picture, the agent was always shown on the left side in order to facilitate fast comprehension of the depicted scene. There were always two sentences that potentially matched a given sentence (i.e., a SVO and an OSV sentence for each picture). For instance, the left picture shown in **Figure 1** was used as target picture for sentence SVO I and OVS II in **Table 1**. All pictures were presented to the participants before they performed the audio-visual picture matching paradigm to familiarize them with the visual stimuli. All pictures are publicly available^1^.

### Audio-Visual Picture-Matching Paradigm

**FIGURE 2:**
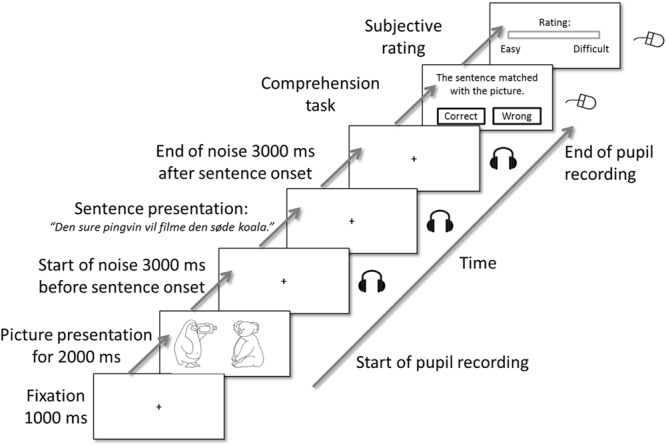
**Trial structure of the audio-visual picture-matching paradigm.** Participants saw a picture on screen for 2000 ms, followed by a visual fixation cross and a simultaneous acoustical presentation of a sentence in background noise. Background noise was presented 3000 ms before and ended 3000 ms after sentence offset. After the acoustic presentation, participants’ task was to decide whether the picture matched with the sentence or not. Pupil dilations were measured from the picture onset until the participants’ response in the comprehension task. The comprehension task was followed by a subjective rating of the experienced difficulty.

The trial procedure for the audio-visual picture matching paradigm is shown in **Figure 2**. After an initial silent baseline showing a fixation cross (for 1 s), the participants were shown a picture (either target or competitor) for a period of 2 s. This was followed by a 3-s long background noise baseline after which a sentence was presented in the same background noise. After the sentence offset, the background noise continued for additional 3 s. A fixation cross was presented during the sound stimulus presentation. After the final noise offset, the participants were prompted to decide whether the sentence matched the picture or not via a button press (left or right mouse button). After the comprehension task, the participants were instructed to rate how difficult it was to understand the sentence using a continuous visual analog scale (McAuliffe et al., 2012). They were asked to indicate their rating by positioning a mouse on a continuous slider marked “easy” and “difficult” at the extremes.

First, the participants performed one training block, which contained 10 trials. After training, each participant listened to 159 sentences, divided into two blocks. Both SVO and OVS sentences were presented in a lower-level noise condition (+12 dB SNR) or in a higher-level noise condition (-6 dB SNR). The noise masker was a stationary speech-shaped noise with the long-term frequency spectrum of the speech. Filler trials were included were the picture either did not match the character or the action of the spoken sentences.

### Cognitive Tests

At the end of the test session, the participants performed two cognitive tests: a digit-span test and a reading span task. The digit span test was conducted in a forward and a backward version. The forward version is thought to primarily asses working memory size (i.e., number of items that can be stored) whereas the backward version reflects the capacity for online manipulation of the content of working memory (e.g., Kemper et al., 1989; Cheung and Kemper, 1992). In the forward version, a chain of digits was presented aurally and the participants were then asked to repeat back the sequence. In the backward version, the participants were asked to repeat back the sequence in reversed order. To calculate the scores for the digit span test, one point was awarded for each correctly repeated sequence (according to the traditional scoring; see Tewes, 1991). The scores were presented in percentages correct, i.e., how many sequences out of the 14 sequences were repeated correctly. In addition, while the participants performed the digital span tests, pupil dilations were recorded to obtain a physiological correlate of effort.

In the reading span task, the participants were presented with sequences of sentences on the screen and instructed to determine, after each sentence, whether the sentence made sense or not (Daneman and Carpenter, 1980). After each sentence, a letter was presented on the screen and the participant was asked to remember the letter. After a set of sentences (length of the set varied between 3 and 11 sentences), the participant was prompted to recall the letters presented between sentences. The number of letters that were correctly recalled were scored regardless of the order in which they were reported. The reading span score was defined as the aggregated number of letters correctly recalled across all sentences in the test. Letters were used as targets rather than sentence words in order to make the task less reliant on reading abilities.

### Apparatus

The experiment was performed in a sound-proof booth. Participants were seated 60 cm from the computer screen and a chin rest was used to stabilize their head. Visual stimulus was presented on a 22″ computer screen with a resolution of 1680 × 1050 pixels. The stimuli were delivered through two loudspeakers (ADAM, A5X), located next to the screen. An eye-tracker system (EyeLink 1000 desktop system, SR Research Ltd.) was used to record participants’ pupil dilation with a sampling rate of 1000 Hz throughout the experiment. The eye-tracker was calibrated at the beginning of the experiment using a nine-point fixation stimulus. During each trial, pupil size and pupil x- and y-traces were recorded for detecting horizontal and vertical eye-movements, respectively. The eye tracker sampled only from the left eye.

### Pupil Data Analysis

**FIGURE 3:**
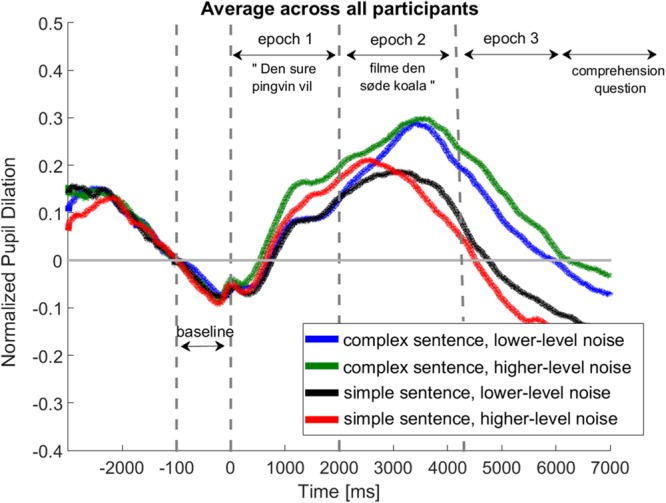
**Normalized pupil dilation averaged across all participants for all four conditions.** Time axis starts with the onset of sentence presentation. Horizontal lines indicated interval used for baseline correction and the different epochs in which the mean pupil response was calculated.

The recorded data were analyzed for 20 participants in a similar way as reported in previous studies (Piquado et al., 2010; Zekveld et al., 2010, 2011)^2^. First, eye-blinks were removed from the recorded data by classifying samples for which the pupil value was below 3 standard deviations of the mean pupil dilation. After removing the eye-blinks, linear interpolation was applied starting 350 ms before and ending 700 ms after a detected eye-blink. Trials for which more than 20% of the data required interpolation were removed from the further data analysis. For one participant more than 50% of the trials required interpolation and, therefore, this participant was excluded from the further data analysis (Siegle et al., 2003). The data of the de-blinked trails were smoothed by a four-point moving average filter. In order to control for individual differences in pupil range, each trial data point was subtracted by the minimum pupil value of the entire trial time series (from trial onset of the picture presentation until the comprehension task) for each individual participant. Afterward, the pupil data were divided by the range of the pupil size within the entire trial. Finally, the pupil data were normalized by subtracting a baseline value which was defined as the averaged pupil value across 1 s before sentence presentation (when listening to noise alone, see **Figure 3**). The pupil responses were averaged across all participants for each condition. Averaged pupil data were analyzed within three different time epochs (see **Figure 3**). Epoch 1 describes the time from the start of the sentence until the point of disambiguation. Epoch 2 is defined as the time after the point of disambiguation until the sentence offset. Epoch 3 defines the 3 seconds following the sentence offset when the participants are asked to retain sentences in memory until the comprehension question.

## Results

### Speech Comprehension in the Audio-Visual Picture-Matching Task

#### Comprehension Accuracy

**FIGURE 4:**
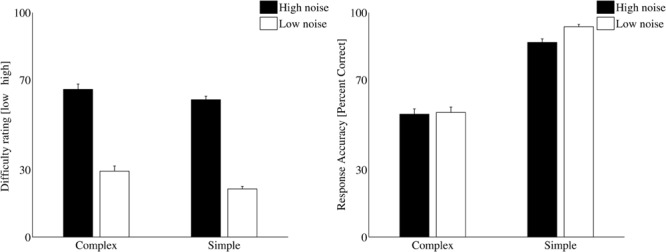
**(Left)** Subjectively rated difficulty averaged across participants for complex (OVS) and simple (SVO) sentence structures presented at higher-level noise (black) and lower-level noise (white) conditions. **(Right)** Response accuracies averaged across all participants and trials for complex (OVS) and simple (SVO) sentences presented at the higher-level noise (black) and the lower-level noise (white) conditions. The error bars show the standard errors.

**Figure 4** shows the mean response accuracy across participants in the audio-visual picture-matching paradigm. The highest accuracy was found for the SVO sentences (93.1% in the lower-level noise condition and 87.8% in the higher-level noise condition). For the OVS structure, the response accuracy was between 57.2% (in the higher-level noise condition) and 58.1% (for the lower-level noise condition). The comprehension accuracy was analyzed using two separate repeated-measures analyses of variance (ANOVA) with complexity (simple, complex) and noise level (high, low) as within-subject factors. The ANOVA revealed a main effect of *complexity* [*F*_(1,18)_ = 15.8, *p* = 0.001, ω = 0.53] showing that the processing of OVS sentences resulted in more comprehension errors compared to the processing of SVO sentences. No effect of the *noise level* on the accuracy scores was found [*F*_(1,18)_ = 1.8, *p* = 0.2] indicating that speech intelligibility was high in both noise conditions.

#### Subjective Ratings

Averaged subjective ratings across all participants were calculated for each condition. The subjective ratings were analyzed using two separate repeated-measures ANOVA with *complexity* and *noise level* as within-subject factors. The ANOVA revealed a main effect of *noise level* [*F*_(1,18)_ = 56.3, *p* < 0.001, ω = 0.779] indicating that the higher-level noise condition was rated as being more difficult compared to the lower-level noise condition. In addition, a small but significant effect of *complexity* on rating was also found [*F*_(1,18)_ = 4.6, *p* = 0.048, ω = 0.223].

#### Time-Averaged Pupil Dilation

**FIGURE 5:**
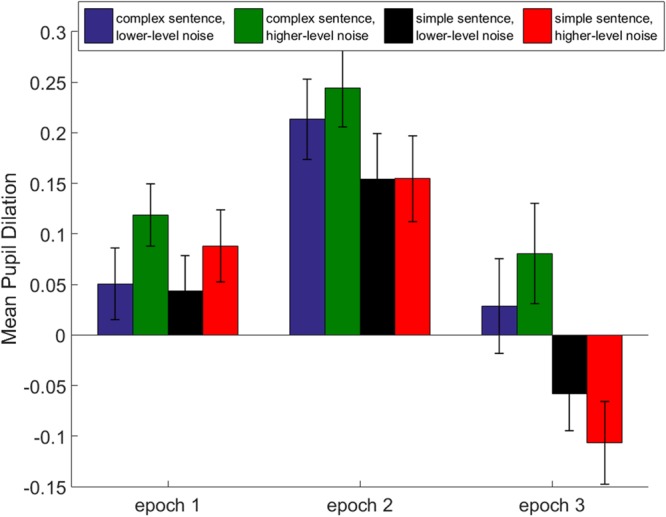
**Mean pupil dilation observed for all four conditions.** Time-averaged pupil dilation was calculated for three different epochs. Epoch 1 is the time when the first part of the sentence was presented. Epoch 2 includes the time after the sentence was disambiguated until the comprehension question. The third epoch is defined as the time from sentence onset until participants’ response. The error bars show the standard deviations.

Averaged pupil dilations across all participants were calculated for each epoch (see **Figure 5**). The dilations were analyzed using separate repeated-measures ANOVA treating *complexity* and *noise level* as within-subjects factors. Separate ANOVAs were performed for each epoch. In epoch 1, there was a significant effect of *noise level* on the time-averaged pupil dilation [*F*_(1,18)_ = 12.1, *p* = 0.03, ω = 0.41], but no effect of *complexity* was found [*F*_(1,18)_ = 0.93, *p* = 0.35]. In epochs 2 and 3, significant effects of *complexity* [*F*_(1,18)_ = 10.8, *p* = 0.004, ω = 0.39; epoch 3: *F*_(1,18)_ = 12.8, *p* < 0.001, ω = 0.52] were revealed. Furthermore, an interaction of *complexity* and *noise level* was found in epoch 3 [*F*_(1,18)_ = 9.0, *p* = 0.008, ω = 0.35].

### Cognitive Data

**FIGURE 6:**
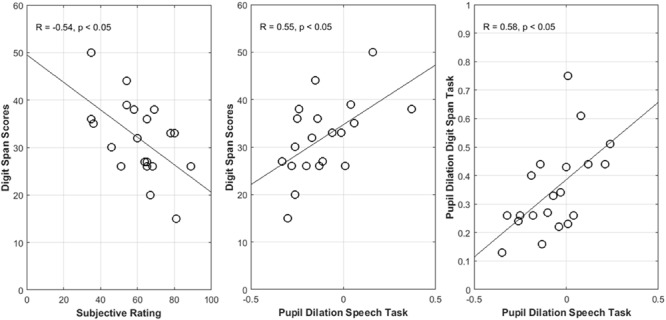
**(Left)** Digit span scores as a function of subjective ratings. **(Middle)** Digit span scores as a function of the of the pupil dilation in the audio-visual picture-matching paradigm. **(Right)** Pupil dilations in the digit span test as a function of the pupil dilation in the audio-visual picture-matching paradigm.

Pearson correlation coefficients between the subjective ratings and the performance in the cognitive tests [digit span forward (DF) score, digit span backward (DB) score, and reading span (RS) score in **Table 2**] were computed. A statistically significant correlation between the subjectively rated effort and the DB score was found for the SVO sentences presented at the higher noise level (*p* < 0.05, see **Table 2** and **Figure 6**).

**Table 2:** Correlation coefficient between the span tests and both the mean pupil dilation in the audio-visual picture matching paradigm and the subjective ratings for all four condition.

	Condition	DB Score	DF Score	RS Score	DB Pupil	DF Pupil
**Mean pupil dilation**	*Simple sentence, Lower-level Noise*	0.22	-0.19	-0.17	**0.58** (*p* = 0.005)	0.15
	*Simple sentence, Higher-level Noise*	**0.55** (*p* = 0.013)	0.08	0.09	-0.12	0.18
	*Complex sentence, Lower-level Noise*	0.15	-0.20	-0.26	0.01	0.15
	*Complex sentence, Higher-level Noise*	0.38	-0.04	-0.01	-0.04	0.46
**Subjective ratings**	*Simple sentence, Lower-level Noise*	0.00	0.12	0.35	0.34	-0.38
	*Simple sentence, Higher-level Noise*	**-0.54** (*p* = 0.013)	-0.13	-0.35	-0.10	-0.08
	*Complex sentence, Lower-level Noise*	0.21	-0.19	0.26	-0.02	0.13
	*Complex sentence, Higher-level Noise*	-0.33	-0.25	-0.29	-0.44	0.22

*DB, digit span backward; DF, digit span forward; RS, reading span; Scores, scores in the span tests; Pupil, averaged pupil response in the digit span backward tests. Bolded values indicate significant correlation coefficients.*

In addition, Pearson correlation coefficients between the mean pupil response in epoch 3 and the performance in the cognitive tests were computed. Significant correlations were only found between the DB score and the pupil dilations in epoch 3 (*p* < 0.05, see **Table 2**), indicating that participants with higher DB scores had larger pupil dilations in the speech task.

Finally, correlations between the pupil dilations in the digit span test and the pupil dilations in the speech task (during epoch 3) were calculated. Pearson correlation coefficients revealed statistical significance (see **Figure 6**; *p* < 0.05), i.e., participants with enlarged pupil dilations in the speech task also showed higher pupil dilations in the span test.

## Discussion

### Effects of Stimulus-Related Factors on Effort

A small but significant increase in pupil dilation due to the increased noise level was found in epoch 1, i.e., while the participants were listening to the first part of the sentence. The changes in pupil dilation due to the noise level were similar for both sentences structures, i.e., independent of the syntactic complexity. Moreover, a clear effect of the noise level on the perceived effort was found, i.e., the listeners reported speech processing as being more effortful when the sentences were presented at lower SNRs. These results are in line with studies that reported changes in pupil dilation and subjective ratings to be dependent on the SNR (Rudner et al., 2012). Zekveld et al. (2010, 2014) observed that pupil dilations and subjective ratings of effort increased with decreasing SNR. However, the current findings also clearly indicate an effect of noise level on effort in listening situations even when speech intelligibility is high. As reflected by the performance in the comprehension task, the participants were able to perform the task equally well at low and high noise levels. To the authors’ knowledge, this is the first study demonstrating that effort increases with decreasing SNR even when speech intelligibility is still high.

Higher processing effort due to the increased syntactic complexity was expected for the OVS sentences compared to the syntactically less complex SVO sentences. Syntactic complexity came into play in epoch 2 when the participants listened to the second part of the sentence. In epochs 2 and 3, the participants were required to process and interpret the sentence by mentally assigning the grammatical roles of agent and patient and matching the spoken sentence content with the scene depicted in the picture. A pupil enlargement was measured for the OVS sentences during epoch 2 and during the retention interval in epoch 3. These findings are consistent with other studies that showed increased effort while processing syntactically complex sentences (Piquado et al., 2010; Wendt et al., 2014). For example, Piquado et al. (2010) reported significantly larger pupil dilations during the retention of complex sentences. In the current study, the sentences were presented in noise in order to test the combined effects of sentence complexity and background noise level. The pupil data demonstrated distinct effects of noise and sentence complexity during epochs 1 and 2. Whereas a main effect of noise was observed in epoch 1, increased pupil dilations induced by the complexity of the sentence were measured in epoch 2. These results suggest that an increased processing effort due to an increased noise level occurs only if the sentence complexity is irrelevant for the task. As soon as the listeners start to process and retain syntactically complex information, the effect of the noise becomes negligible. Interestingly, an interactive effect of noise and complexity on the pupil dilation was found in epoch 3. This interaction was characterized by a steep decrease of the pupil response in the acoustically challenging listening situation (see epoch 3 in **Figure 3**). In other words, although a high pupil size induced by the noise level was detected in epoch 1, the pupil size decreased faster back to the baseline value in the retention period in epoch 3. This observation may suggest that listeners were able to recover faster from the high processing demand in the acoustic more challenging listening situation. However, this fast recovery occurred only for the simple sentence structures. When processing more linguistically complex sentences, this interactive effect was not found.

Whereas the pupil response indicated a clear impact of syntactic complexity, the effect of complexity on the subjective ratings was rather small. This suggests that subjective ratings and pupil dilations reflect different aspects of effort involved in speech comprehension. The pupil responses, interpreted as a physiological correlate of processing effort, were mainly sensitive to the syntactic complexity during sentence comprehension but were not indicative of the subjectively perceived effort. The perceived effort, in contrast, was more influenced by the degradation of the speech signal resulting from the increased background noise level. This is consistent with previous studies also suggesting that potentially different aspects of the effort may be measured when testing different methods and measures of effort (e.g., McGarrigle et al., 2014).

### The Influence of Listener-Related Factors on Effort

In the present study, different span tests were used to measure cognitive abilities of the participants. A moderate correlation between the digit span scores and the pupil dilations was found (**Figure 6**). Higher scores in the backward digit span test were found to correlate with higher pupil dilations in the speech comprehension task in the higher-level noise condition.

This could indicate that individuals with higher WMC allocate and engage more cognitive resources compared to individuals with smaller WMC. Previous studies have also reported higher pupil enlargement during speech processing for individuals with higher scores in cognitive tests (e.g., Zekveld et al., 2011). The results thus are consistent with the notion that individuals with higher cognitive capacities mobilize more working memory resources in acoustical challenging conditions as stated by the resource hypothesis (van der Meer et al., 2010). It is noticeable that significant correlations between WMC and pupil dilations were found specifically in epoch 3 comprising the retention period. This could suggest that pupil dilations specifically indicate the mobilization of working memory resources while storing speech information (and preparing for the upcoming comprehension task). Interestingly, significant correlations appeared only when processing sentences in the acoustically more challenging condition, suggesting that pupil response may further relate to the ability of listeners to rely on some form of working memory processing for compensating increased demands due to challenging acoustics. However, further research is needed to specifically explore these mechanisms.

Interestingly, the subjective ratings were found to be negatively correlated with WMC such that participants with a higher WMC tended to report lower perceived effort when processing SVO sentences in the higher-level noise condition. This suggests that individuals with greater WMC are able to use their resources to cope with the acoustically degraded speech signals and therefore report less effort, as argued by the efficiency hypothesis and the ELU model (Rönnberg et al., 2013). The presented data indicate that the relationship between individual WMC and effort depends on the employed measure. While listeners with a larger memory capacity may engage more resources, as indicated by increased pupil responses, this is not perceived as being effortful. Predictions made by the resource hypothesis with regard to processing effort (and its pupil response correlate) may be interpreted in terms of engagement of enhanced WMC, but not in terms of perceived effort. Predictions about effort made by the ELU model may be interpreted in terms of the subjective experience of effort.

Significant correlations between effort (both perceived effort and processing effort) and the digit span scores were only measured for the SVO sentences in the higher-level noise condition. This indicates that the WMC was only relevant when the induced demands increased due to the acoustic degradation of the speech signal. For the OVS sentences, no correlations between the digit span scores and the rated effort were found both in the higher-level noise and the lower-level noise condition. This suggests that the effort reached a plateau in situations when the cognitive resources could not compensate for the increased processing demands any longer (Johnsrude and Rodd, 2015). Thus, it may be that the available cognitive resources are exhausted when processing OVS sentences, which would further explain why no correlation between the digit span scores and effort were found in neither the higher-level noise nor the lower-level noise condition. No correlations were found between the reading span and the pupil response in the speech task. Note, however, that the procedure for the reading span test differed to the procedure applied in more recent studies (e.g., Lunner, 2003; Rönnberg et al., 2014; Petersen et al., 2016). A revised procedure of the reading span test was developed to include having to remember either the first or the last word of each sentence in the list (see e.g., Lyxell et al., 1996). Since the participants do not know beforehand whether it will be the first or the last word, this revised procedure is suggested to increase the task difficulty and, therefore, the reading span score is supposed to reflect a more sensitive measure of the WMC. Thus, the missing correlation between the reading span score and the speech task might be explained by the procedure applied in the current study.

In this study, WMC was considered as a listener-related factor that potentially influences effort. However, there may be other listener-related factors that have not been considered in the current study. Interestingly, positive correlations between the pupil dilations in the digit span test and the pupil dilations in the speech task were found (**Figure 6**). Listeners that allocated more resources in the speech task also tended to mobilize more resources in the digit span test. This may indicate that some listeners generally engaged more resources than others when performing a task. Other potential listener-specific factors affecting effort have been discussed in the literature. For instance, the level of motivation of individual participants could further influence the intensity of effort mobilization (Brehm and Self, 1989; Gendolla and Richter, 2010). With increasing success importance or with increasing motivation intensity, the amount of effort involved in a task can increase. It is possible that those participants who showed increased pupil dilations in both tasks were more motivated than those who exhibited smaller pupil dilation in both tasks. However, since motivation and success importance were not tested in the present study, the potential contribution of motivation to the results from this study remains to be clarified.

### Implications for Future Research

The audio-visual picture-matching paradigm presented in this study is well suited for studying speech processing in realistic communication situations. Monitoring increased effort during speech processing when intelligibility is high is crucial since it indicates challenges that constantly appear in everyday life. Moreover, in order to perform the task, listeners need to conduct a syntactic analysis of the sentence. This is in contrast to many speech intelligibility studies where the participants are typically asked to repeat back the recognized words of a sentence (Hagerman, 1984; Plomp, 1986). However, repetition does not necessarily involve any processing of the sentence structure or meaning that may constitute an important component of the challenges experienced in every day speech comprehension.

Extensive engagement of cognitive resources in everyday speech processing may eventually lead to fatigue or tiredness. Previous research suggested that hearing-impaired listeners are particular challenged in adverse conditions both with regard to speech perception performance and in terms of their effort required to achieve successful speech perception (Plomp, 1986; Rönnberg et al., 2013; Wendt et al., 2015). Consequences of increased effort can be, for example, a higher level of mental distress and fatigue leading to stress (Gatehouse and Gordon, 1990; Kramer et al., 2006; Edwards, 2007; Hornsby, 2013). Since traditional speech recognition tests are not sensitive to detect changes in effort in more realistic communication situations, there seems to be a need for new methods and measures to examine effort for hearing-impaired people. The findings of the present study suggest that pupil responses and subjective ratings are independent measures addressing different aspects of effort. Thus, when testing one measure of effort, the other measure is not necessarily reflected. This should be taken into account by researchers and clinicians when applying either one or the other method in their studies (McGarrigle et al., 2014).

## Summary and Conclusion

Three main observations were made in the present study. First, effects of increased demands due to background noise level and syntactic complexity were reflected in both the subjective ratings and pupil dilations. Second, the interaction between background noise level and syntactic complexity was rather small. Instead, separable effects of noise level and complex syntax on the subjective ratings and the pupil dilations were found: Increased syntactical complexity resulted in enlarged and prolonged pupil dilations, whereas a higher background noise level resulted in the task being rated as more effortful. Third, individual differences in cognitive abilities of the participants correlated differently with perceived effort and processing effort. Participants with higher scores in the backward span test (indicating higher WMC) showed increased pupil dilations but also reported the speech task to be less effortful than participants with lower scores. Overall, these findings demonstrate that pupil dilations and subjectively rated effort can vary in situations when intelligibility is at a high level and represent different aspects of effort. The methods and measures employed to investigate effort therefore need to be chosen carefully depending on the specific research question and hypothesis.

## Author Contributions

The author DW developed the conception and design of the study, supervised the data acquisition, analyzed, and interpreted the data, and wrote the paper. DW gives the final approval of the version to be published, and agrees to be accountable for all aspects of the work. The author TD was involved in developing the design of the study, enabled the data acquisition, and substantial contributed to the interpretation of the data. TD was involved in writing and critically revising this version of this manuscript for important intellectual content. TD gives the final approval of the version to be published, and agrees to be accountable for all aspects of the work. The author JH developed the conception and design of the study, supervised the data acquisition, analyzed, and interpreted the data. JH was involved in writing and critically revising this version of this manuscript for important intellectual content. JH gives the final approval of the version to be published, and agrees to be accountable for all aspects of the work.

## Conflict of Interest Statement

The authors declare that the research was conducted in the absence of any commercial or financial relationships that could be construed as a potential conflict of interest.

## Footnotes

1 http://www.aulin.uni-oldenburg.de/49349.html

2 All data exclusions, all manipulations, and all measures were reported in this study.
